# Arms and ammunitions: effectors at the interface of rice and it’s pathogens and pests

**DOI:** 10.1186/s12284-021-00534-4

**Published:** 2021-11-18

**Authors:** Sohini Deb, Vishnu Narayanan Madhavan, C. G. Gokulan, Hitendra K. Patel, Ramesh V. Sonti

**Affiliations:** 1 CSIR-Centre for Cellular and Molecular Biology (CSIR-CCMB), Hyderabad, 500007 India; 2 Present Address: Department of Plant and Environmental Sciences, University of Copenhagen, 1871 Frederiksberg C, Denmark; 3 Present Address: Indian Institute of Science Education and Research (IISER) Tirupati, Tirupati, 517507 India

**Keywords:** Rice, Effectors, Immunity, Pathogen, Pest, Disease

## Abstract

The plant immune system has evolved to resist attack by pathogens and pests. However, successful phytopathogens deliver effector proteins into plant cells where they hijack the host cellular machinery to suppress the plant immune responses and promote infection. This manipulation of the host cellular pathways is done by the pathogen using various enzymatic activities, protein- DNA or protein- protein interactions. Rice is one the major economically important crops and its yield is affected by several pathogens and pests. In this review, we summarize the various effectors at the plant- pathogen/ pest interface for the major pathogens and pests of rice, specifically, on the mode of action and target genes of the effector proteins. We then compare this across the major rice pathogens and pests in a bid to understand probable conserved pathways which are under attack from pathogens and pests in rice. This analysis highlights conserved patterns of effector action, as well as unique host pathways targeted by the pathogens and pests.

## Background

The growing global population necessitates increased food production even as resources such as water and land are becoming limiting and environmental concerns dictate lesser use of inputs such as fertilizers and pesticides. To ensure food security and sustainable agricultural practices, the development of newer crop varieties is necessary. This involves addressing various aspects, such as yield and tolerance to biotic and abiotic stresses. The biotic stresses include plant diseases caused by bacteria, fungi and viruses as well as damage caused by nematodes and insect pests. Understanding the molecular intricacies of these plant-pathogen/pest interactions can be an important aid in developing disease tolerant plant varieties. A major role in the success of these pathogens and pests is played by the class of molecules, known as “effectors”. Effectors secreted by pathogens/pests can function in gaining entry into the plant, obtaining access to its nutrients, to suppress host defense responses and to eventually multiply in or on the plant. These effectors can either be proteins or metabolites. Because of their importance in promoting infection/infestation, a better understanding of effector biology can potentially help in conceptualizing newer strategies for developing biotic stress tolerant plant varieties. Numerous reviews have extensively covered effector biology from the perspective of the pathogens (Franceschetti et al. 2017; Dean et al. 2012; Toruno et al. 2016; Varden et al. 2017; Dou and Zhou 2012). This article aims to review effectors deployed by the pathogens and pests of rice and identify any common strategies that they may be targeting.

*Oryza sativa*, or rice, is the staple food for nearly half of the global population and is an economically important crop across nations (Khush 2005). Its production is constantly threatened by many different diseases/pests. On an average, farmers lose an estimated 37% of their rice crop to diseases and pests every year (http://www.knowledgebank.irri.org). Various pathogens and pests have been described in rice, although the biology of their effectors has been explored only in a few of the major pathogens. Two members of the bacterial genus *Xanthomonas* cause the serious bacterial blight (BB) and bacterial leaf streak (BLS) diseases. *Magnaporthe oryzae* causes blast of rice and is a well-established fungal disease model in rice. Other emerging rice- pathogen disease models among filamentous pathogens include the fungus *Rhizoctonia solani* and *Pythium* oomycetes species. About 20 species of insects are known to cause significant economic damage in rice (http://www.knowledgebank.irri.org). Some important pests of the rice plant include brown plant hopper (BPH), gall midge and yellow stem borer.

Research on rice-pathogen/pest interaction at the molecular level is a very active field and warrants more investigation. This review seeks to highlight the information available for rice in a comprehensive manner, also emphasizing on the need for further characterisation of the host targets of effectors secreted by  pathogens and pests.

## Main Text

### Bacterial Pathogens: Microscopic but Devastating

**Fig. 1:**
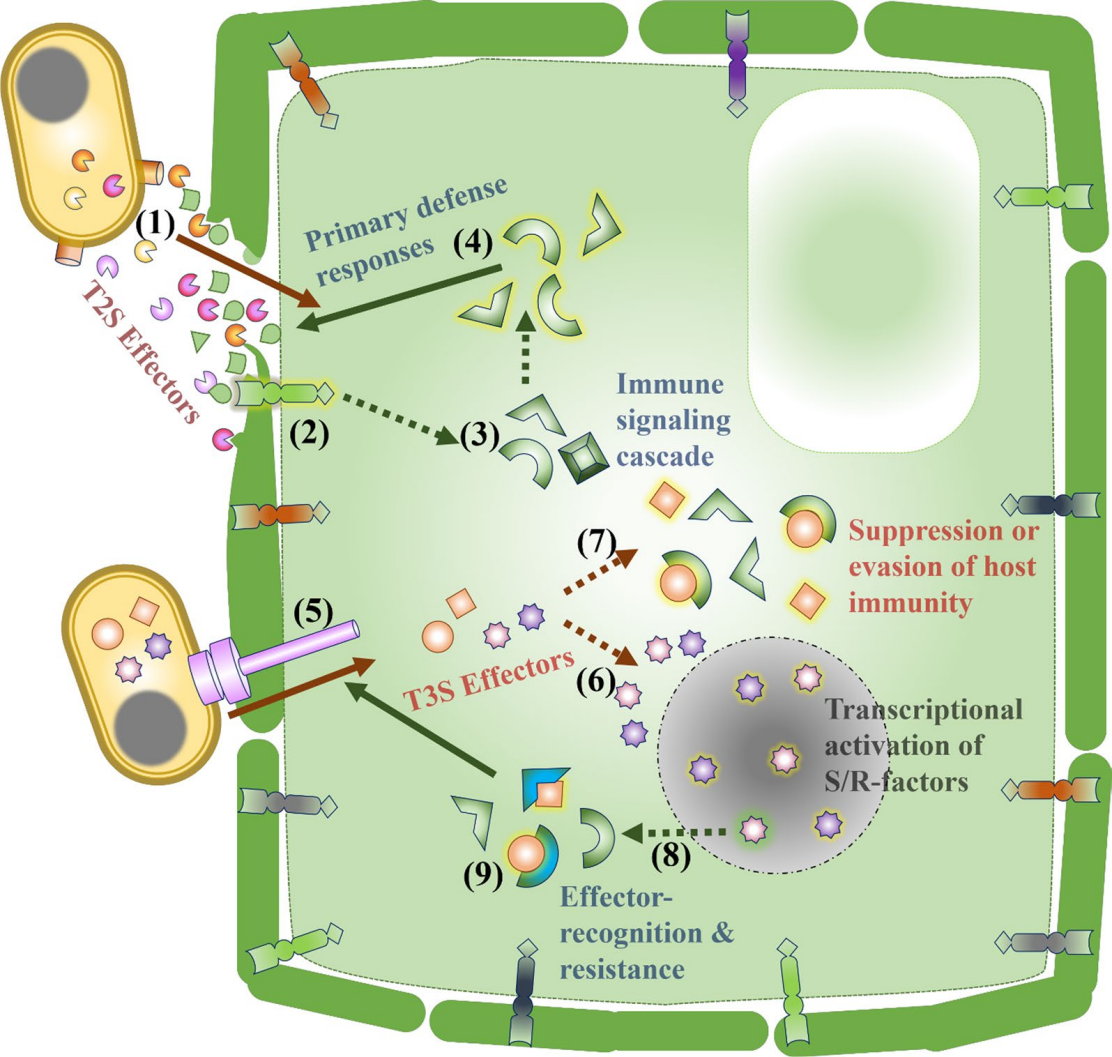
Overview of rice-*Xoo* interaction with a focus on effectors during pathogenesis. The *Xoo*-rice interaction is an example of a complex multi-layered arms race between the pathogen and host with effectors playing remarkable roles in determining the pathogenicity. *Xoo* gains access to the plant cellular contents through digesting the cell wall. This is achieved via secreting an array of cell wall degrading enzymes (CWDEs, shown as different coloured pie shapes) through the type II secretion system (1). The damage-associated molecular patterns (DAMPs) from degradation products of CWDEs and pathogen-associated molecular patterns (PAMPs) are sensed by specific receptors at the plasma membrane (2). This activates downstream signalling cascades (3) such as MAPK signalling leading to activation of transcription factors and upregulation of defense genes, resulting in defense responses such as callose deposition, programmed cell death, and release of ROS (4). The effector proteins secreted via the type III secretion system are directly delivered into the plant cell cytoplasm (5). These effectors consist of transcription activator-like (TAL) effectors, which are DNA binding proteins that upregulate plant genes leading to further susceptibility (6) (Classical example is *SWEET* gene upregulation in *Xoo*-rice interaction). Another class of T3S effectors—non-TAL effectors -are involved in dampening the immune responses by targeting defense signalling pathways, working directly or indirectly by binding to plant proteins (7). The plant counters these effectors using multiple mechanisms. This involves the executor R genes whose transcription is activated by TAL effectors leading to strong immune response and thus resistance (8), and by resistance proteins that target effectors directly or indirectly (9)

*Xanthomonas* includes a large group of plant pathogenic Gram- negative bacteria which infect more than 200 different plant species (Boch and Bonas 2010; Buttner and Bonas 2010). Depending on the host range, and symptomology on a host, they have been grouped into different pathovars (pv.) (Dye et al. 1980). The primary mode of entry for *Xanthomonas* bacteria into rice plants are natural openings like stomata and hydathodes. *X. oryzae* pv*. oryzae* (*Xoo*) causes bacterial blight (BB) and *X. oryzae* pv. *oryzicola* (*Xoc*) causes bacterial leaf streak (BLS) in rice. Infection sites are characterised by water-soaked lesions and chlorosis, and often become necrotic. *Xoo* and *Xoc* use effectors secreted through different types of protein secretion systems, such as the type II secretion system (T2SS) and the type III secretion system (T3SS) (Fig. 1). Effectors can thus be divided into two broad groups: those acting in the extracellular spaces of host tissues (apoplastic) or those acting within host cells (cytoplasmic) (Carella et al. 2018). Apoplastic effectors are secreted via the T2SS of bacterial pathogens (Chang et al. 2014). These molecules are typically involved in the enzymatic degradation of plant cell walls, immune evasion, or the suppression of host proteolytic activity (Toruno et al. 2016; Wang and Wang 2018). The cell wall degrading enzymes secreted by *Xoo* serve to breach the cell wall, but the damage that they cause also triggers host immune responses (Fig. 1). To suppress and evade host immune responses, *Xoo* secretes effector proteins into plant cells via its T3SS. The T3SS apparatus is a needle-like structure spanning both the bacterial membranes which injects the effectors directly into the plant cell (Weber et al. 2005). Hence these effectors are termed as “cytoplasmic effectors”, their site of action being inside the plant cell (Khan et al. 2018). *Xanthomonas* type III effector proteins are classified either as Transcription activator-like (TAL) effectors which have a DNA binding domain or non-TAL effectors (also known as *Xanthomonas* outer proteins or Xops) which lack the same (Buttner and Bonas 2010; White and Yang 2009).

### Apoplastic Effectors: The Two-Edged Swords

As part of its virulence strategy, *Xoo* secretes a battery of plant cell wall– degrading enzymes (CWDEs) using its T2SS (Jha et al. 2005, 2007). The *Xoo* genome contains a single gene cluster encoding for proteins of the type II secretion system (Lee et al. 2005). The T2SS secreted CWDEs are important virulence determinants of the pathogen (Ray et al. 2000; Tayi et al. 2016a, 2016b; Rajeshwari et al. 2005). Action of these CWDEs on the rice cell wall results in damage that is sensed by the host and leads to induction of immune responses.

Proteins secreted by the T2SS include CWDEs such as xylanase (Ray et al. 2000; Rajeshwari et al. 2005; Qian et al. 2013), cellulase/endoglucanase (Sun et al. 2005; Furutani et al. 2004), putative cysteine protease (Furutani et al. 2004), cellobiosidase (Tayi et al. 2016a, 2018), lipase/esterase (Aparna et al. 2009), an extracellular protease EcpA (Zou et al. 2012), endoglucanase EglXoB (Hu et al. 2007), etc. A number of these CWDEs have been shown to be required for full virulence on rice and some of them have also been shown to be involved in eliciting host immune responses *in planta* (Tayi et al. 2016a; Jha et al. 2005). These immune responses are further suppressed by the type III secreted effectors, or the cytoplasmic effectors.

### Cytoplasmic Effectors: The Tale of the TALEs

The cytoplasmic effectors consist of TAL effector proteins (TALEs) and non-TAL effector proteins. The TAL effector proteins enter the nucleus and execute their role as transcription factors by activating the expression of plant susceptibility genes (Boch and Bonas 2010). TAL effector family proteins typically consist of an N-terminal secretion signal and a variable number of near- identical repeats of a 34–amino acid sequence (Mudgett 2005; Bonas et al. 1989; Hopkins et al. 1992). They also have at least one nuclear localisation signal (NLS), and an acidic activation domain (AAD) at the C- terminus (Gurlebeck et al. 2006). Both *Xoo* and *Xoc* express a large number of TAL effectors, exceeding eight in *Xoo* isolates and over twenty in *Xoc* isolates (Wilkins et al. 2015; Salzberg et al. 2008; Bogdanove et al. 2011). Some of the most conserved TAL effectors genes are *avrXa7*, *pthXo1*, *pthXo2* and *pthXo3* (Yang and White 2004). Loss of these four effectors from *Xoo* results in highly reduced virulence and affects symptom development (Bai et al. 2000; Yang and White 2004). The target genes of these effectors are commonly referred to as susceptibility genes. Mutations in the promoters of these genes render these host genes non-responsive to TAL effectors and they function as recessive resistance genes. An alternate response of the plant is a strong suppression of disease development in response to diverse TAL effectors, such as what is mediated by the *Xo1* resistance locus (Triplett et al. 2016). The target genes of the TAL effectors are a diverse class of genes, the major targets being transcription factors, receptor kinases or *SWEET* genes (Mücke et al. 2019).

The well- characterised *R-*genes that confer tolerance against *Xoo* are *Xa1*, *xa5*, *Xa7*, *Xa10*, *xa13*, *Xa21*, *Xa23*, *Xa27,* and *Xa3/Xa26* (Song et al. 1995; Yoshimura et al. 1998; Iyer and McCouch 2004; Sun et al. 2004; Chu et al. 2006b; Gu et al. 2005; Xiang et al. 2006; Wang et al. 2015; Tian et al. 2014; Chen et al. 2021) (Table 1). The *Xa21* gene encodes a leucine rich repeat (LRR)-type receptor kinase and interacts with the E3 ligase XB3 (Xa21 Binding Protein 3) (Wang et al. 2006; Song et al. 1995). The elicitor of Xa21 mediated resistance is a sulphated-tyrosine containing peptide secreted by *Xoo* called RaxX (da Silva et al. 2004; Shen and Ronald 2002; Burdman et al. 2004; Pruitt et al. 2015). *xa13* encodes a plasma membrane protein (Chu et al. 2006b), *Xa1* encodes a nucleotide-binding site–LRR protein (Yoshimura et al. 1998) and *Xa3/Xa26* encodes an LRR receptor kinase-like protein (Sun et al. 2004; Xiang et al. 2006). Another class of R-genes, called executor R-genes, have also been cloned and characterised. This class  include the genes *Xa7, Xa10, Xa23,* and *Xa27*. Common features of these genes are (i) their expression is observed only in the presence of a cognate TAL effector in the infecting *Xoo* strain, (ii) the gene induction occurs only in resistant cultivars, (iii) induction of these genes results in hypersensitive response (HR) and thus resistance to *Xoo*. *Xa7, Xa10, Xa23,* and *Xa27* were shown to be respectively induced by their cognate TAL effectors and leading to HR and resistance to *Xoo* (Gu et al. 2005; Wang et al. 2015; Tian et al. 2014; Chen et al. 2021). Further, Xa10 has been shown to be localised in endoplasmic reticulum (ER) as a hexamer and could trigger cell death by ER Ca^2+^ depletion via a conserved mechanism (Tian et al. 2014). *Xa7* was shown to be highly induced at high temperature regime (35℃) and is proposed as a suitable source for resistance to *Xoo* considering the global temperature changes. Notably, among all the TAL effectors that induce executor R-genes, only AvrXa7 has been shown to be essential for *Xoo* virulence (Chen et al. 2021).

**Table 1:** TAL effectors of *Xoo*

TAL effector	Target gene	Target gene family	References
TalB	*OsTFX1 OsERF#123*	bZIP transcription factor AP2/ERF transcription factor	Tran et al. (2018)
TalC	*OsSWEET14*	Plasma membrane protein (sucrose transporter)	Streubel et al. (2013)
AvrXa7	*OsSWEET14/ Os11N3* *Xa7*	Plasma membrane protein (sucrose transporter) Executor R-gene (of unknown function)	Antony et al. (2010), Yuan and Wang (2013) and Chen et al. (2021)
AvrXa10	*Xa10*	Trans-membrane protein localised to the endoplasmic reticulum membrane (Executor R-gene)	Tian et al. (2014)
AvrXa23	*Xa23*	Trans-membrane protein (Executor R-gene)	Wang et al. (2014)
AvrXa27	*Xa27*	Executor R-gene (of unknown function)	Gu et al. (2005)
PthXo1	*OsSWEET11/Os8N3*	Plasma membrane protein (sucrose transporter)	Yang et al. (2006)
PthXo2	*OsSWEET13/xa25*	Plasma membrane protein (sucrose transporter)	Zhou et al. (2015)
PthXo3_JXOV_	*CDS1, CDS2, CDS3* *OsSWEET14*	Unknown Plasma membrane protein (sucrose transporter)	Li et al. (2018a)
PthXo3_PXO99A_	*OsSWEET14/Os11N3*	Plasma membrane protein (sucrose transporter)	Antony et al. (2010)
PthXo6	*OsTFX1*	bZIP transcription factor	Sugio et al. (2007)
PthXo7	*OsTFIIAɣ1*	Small subunit of the transcription factor IIA	Sugio et al. (2007)

Many of the TALEs target a class of sugar transporters known as the *SWEET* genes, eg., PthXo1, PthXo6 and PthXo7. The TAL effector PthXo1 binds to the promoter region of *OsSWEET11* (also called *Os8N3* or *Xa13*), which is a sucrose transporter gene to induce its expression and promote bacterial pathogenicity. The rice gene *xa13* is a recessive resistance allele of *Os8N3* (Yang et al. 2006; Antony et al. 2010; Chu et al. 2006a) and is not induced by PthXo1, whereas the susceptible gene *Xa13* is pathogen inducible. This recessive allele, however, can be overcome by strains of *Xoo* producing any one of the type III TAL effectors AvrXa7, PthXo2, or PthXo3. Both AvrXa7 and PthXo3 induce the expression of *Os11N3*/ *OsSWEET14*, another *SWEET* gene which apparently compensates for the inability of *Xoo* to induce *xa13* (Antony et al. 2010; Yuan and Wang 2013). The TAL effector PthXo2 also induces *OsSWEET13* (also known as *xa25* in the rice cultivar Minghui 63) (Zhou et al. 2015). Thus, TALEs target multiple sugar transporters in the *SWEET* gene family, likely facilitating sugar export for bacterial consumption (Chen et al. 2010). This has been directly demonstrated for PthXo2 wherein heterologous expression of its target *OsSWEET13* in *Nicotiana benthamiana* leaf cells elevated sucrose concentrations in the leaf apoplasm (Zhou et al. 2015).

Other targets of *Xoo* TAL effectors include *OsTFX1* and *OsTFIIAɣ1*, the small subunit of the transcription factor IIA (Sugio et al. 2007). The resistant allele of *OsTFIIAɣ5* is encoded by *xa5* (Iyer and McCouch 2004; Blair et al. 2003). In order to overcome the resistance mediated by *xa5*, PthXo7 is used by the bacteria to increase the expression of *OsTFIIAɣ1* (Ma et al. 2018).

More recently, "truncTALEs," for "truncated TAL effectors", alternatively known as interfering TALEs, or iTALEs, have been described in the *Xoo* strain PXO99A as well as in *Xoc* BLS256, which suppress disease resistance. As compared to typical TALEs, these proteins lack a transcription activation domain and are expressed from genes that were previously considered pseudogenes (Read et al. 2016; Ji et al. 2016, 2020a).

### The *Xoo* Non-TAL Effectors

In *Xoo*, 16 non-TAL effectors were initially identified. Out of these, nine effectors shared homology with previously identified T3S effectors in other plant-pathogenic bacteria whereas seven effectors appeared to be *Xoo* specific (Furutani et al. 2009). Expression of the type III effectors is regulated by genes that regulate the *hrp* cluster (hypersensitive response and pathogenicity), specifically, *hrpG* and *hrpX* (Song and Yang 2010). Many of these effectors, were shown to be required for the full virulence of the strain (Gupta et al. 2015; Song and Yang 2010; Zhao et al. 2013; Mondal et al. 2016).

True to their putative function, the type III effectors were shown to suppress immune responses. XopZ_PXO99A_ suppressed callose deposition induced by treatment of a T3SS^–^ strain (Song and Yang 2010). XopR_Xoo_ enhances the growth of *Xanthomonas campestris* pv. *campestris* T3SS^−^ in *Arabidopsis*, probably by suppression of PAMP (pathogen-associated molecular pattern) -triggered early-defense genes, for example, a leucine-rich repeat protein kinase, a cysteine/histidine-rich C1 domain family protein, Flg22-induced receptor-like kinase 1 (FRK1) and a member of CYP81F, induced by the T3SS^−^ mutant (Akimoto-Tomiyama et al. 2012). Furthermore, XopR_PXO99A_ suppresses PAMP-triggered stomatal closure in transgenic *Arabidopsis* expressing XopR_PXO99A_ (Wang et al. 2016b). Expression of XopP_Xoo_ in rice strongly suppresses peptidoglycan (PGN)- and chitin-triggered immunity and tolerance to *X. oryzae* (Ishikawa et al. 2014). XopQ_BXO43_, as well as XopX _BXO43_ were shown to suppress plant defense responses by targeting 14-3-3 proteins of rice, which are adapter proteins in signalling pathways (Deb et al. 2019, 2020).

Interestingly, these effectors seem to be targeting a varied number of pathways, indicating towards the involvement of these pathways in immune responses (Table 2). XopN_KXO85_ was shown to interact with a thiamine synthase (OsXNP) and OsVOZ2 (a transcription factor) (Cheong et al. 2013). Since treatment with thiamine was shown to enhance resistance to pathogen invasion in rice (Ahn et al. 2005, 2007), XopN seems to suppress immune responses by blocking thiamine synthesis. Another type III non-TAL effector, XopY was shown to inhibit the phosphorylation of the receptor kinase OsRLCK185 and the downstream MAPK signalling, and hence promote pathogenesis (Yamaguchi et al. 2013b). Later it was further shown that this receptor kinase is involved in the perception of both peptidoglycan (PGN) as well as chitin signalling, indicating for its possible involvement in response to bacterial and fungal pathogens (Wang et al. 2017). Another effector which may have a role in interfering with peptidoglycan and chitin induced signalling is the *Xoo* effector XopP_Xoo_, which targets OsPUB44, a rice ubiquitin E3 ligase. XopP_Xoo_ was shown to directly interact with the U-box domain of OsPUB44 and inhibit ligase activity. Silencing of *OsPUB44* suppressed PGN- and chitin-triggered immunity (Ishikawa et al. 2014). On the other hand, XopL itself exhibits E3 ubiquitin ligase activity and interacts with ferredoxin (NbFd), to target it for ubiquitination and ubiquitin-mediated degradation, thereby increasing reactive oxygen species (ROS) production (Ma et al. 2020). XopI has also been shown to act as a F-box adapter and interacts with a thioredoxin protein, OsTrxh2, to strongly inhibit the host's OsNPR1-dependent resistance to *Xoo* (Ji et al. 2020b).

**Table 2:** Non-TAL effectors of *Xoo*

Effector	Localisation	Pathway inhibited/relevant information
XopC	Cytoplasmic (Wang et al. 2016b)	
XopF	Chloroplast (predicted) (Zhao et al. 2013)	
XopG		Suppression of XopQ- XopX mediated immune responses (Deb et al. 2020)
XopI		Acts as a F-box adapter and interacts with a thioredoxin protein, OsTrxh2, to strongly inhibit the host's OsNPR1-dependent resistance to *Xoo* Required for complete virulence in rice (Ji et al. 2020b)
XopL	Cytoplasmic (Wang et al. 2016b)	Exhibits E3 ubiquitin ligase activity and interacts with ferredoxin (NbFd), to target it for ubiquitination and ubiquitin-mediated degradation, thereby increasing reactive oxygen species (ROS) production (Ma et al. 2020)
XopN		Thiamine synthesis (Cheong et al. 2013) Suppresses PGN- triggered MAPK activation (Long et al. 2018)
XopP	Chloroplast (predicted) (Zhao et al. 2013)	Suppresses PGN- and chitin-triggered immunity and resistance and targets OsPUB44, a rice ubiquitin E3 ligase (Ishikawa et al. 2014) Suppression of XopQ- XopX mediated immune responses (Deb et al. 2020)
XopQ	Nucleo- cytoplasmic (Deb et al. 2019)	14-3-3 mediated suppression of rice immune responses (Deb et al. 2019) Required for complete virulence in rice (Gupta et al. 2015)
XopR	Plasma membrane (Zhao et al. 2013)	Receptor kinase interaction (Wang et al. 2016b) Immune response suppression (Akimoto-Tomiyama et al. 2012) Required for complete virulence in rice (Zhao et al. 2013)
XopU		Suppression of XopQ- XopX mediated immune responses (Deb et al. 2020)
XopV	Cytoplasmic (Wang et al. 2016b)	Suppresses PGN- triggered MAPK activation (Long et al. 2018) Suppression of XopQ- XopX mediated immune responses (Deb et al. 2020)
XopW	Cytoplasmic (Wang et al. 2016b)	
XopX	Nucleo- cytoplasmic (Deb et al. 2020)	14-3-3 mediated suppression of rice immune responses (Deb et al. 2020)
XopY		Chitin & PG induced MAPK signalling (Yamaguchi et al. 2013b)
XopZ		Suppresses PGN- triggered MAPK activation (Long et al. 2018)
XopAA		Receptor kinase interaction & Brassinosteroid signalling (Yamaguchi et al. 2013a)
XopAE	Chloroplast (predicted) (Zhao et al. 2013)	
AvrBs2		Suppression of XopQ- XopX mediated immune responses (Deb et al. 2020)

XopAA strongly inhibited host resistance to *X. oryzae*, possibly by interaction with OsBAK1 (BRI1—associated kinase). OsBAK1 interacts with FLS2, the receptor kinase sensor of the PAMP flg22, in the initial steps of its signalling, making it an essential component of signalling responses induced by PAMPs (Chinchilla et al. 2007). OsBAK1 is also a co- receptor of the hormone brassinosteroid (BR) (Wang et al. 2008), suggesting that the virulence promoting activity of XopAA is mediated by the inhibition of OsBAK1 (Yamaguchi et al. 2013a). Similarly, XopR_PXO99A_ was shown to interact with BIK1, a receptor-like cytoplasmic kinase (RLCK) and appears to be phosphorylated by it. BIK1 mediates PAMP-triggered stomatal immunity. In addition, XopR was seen to associate with other RLCKs as well apart from BIK1 and thus may suppress plant immunity by targeting RLCKs (Wang et al. 2016b; Akimoto-Tomiyama et al. 2012).

### Effectors Employed by Filamentous Pathogens of Rice

The filamentous pathogens such as fungi and oomycetes are known to cause devastating plant diseases leading to significant yield losses worldwide. Some of the fungal diseases of rice include rice blast caused by *Magnaporthe oryzae* (*M. oryzae*), rice sheath blight caused by *Rhizoctonia solani*, false smut of rice caused by *Ustilaginoidea virens*, sheath rot of rice caused by *Sarocladium oryzae* and bakanae disease caused by *Gibberella fujikuroi* (Elazegui and Islam 2003). In addition, the oomycete genus *Pythium* is also known to cause diseases in rice (Van Buyten and Hofte 2013).

The filamentous pathogens have evolved a large repertoire of secreted effectors of various functions, which play a major role in disease progression. With respect to rice-filamentous pathogen biology, the most well-studied pathogen is *M. oryzae* (Dean et al. 2012; Pennisi 2010). Thus, the scope of this review would primarily be referring to the effectors of *M. oryzae*.

### The ‘Blast’ by *Magnaporthe oryzae*

**Fig. 2:**
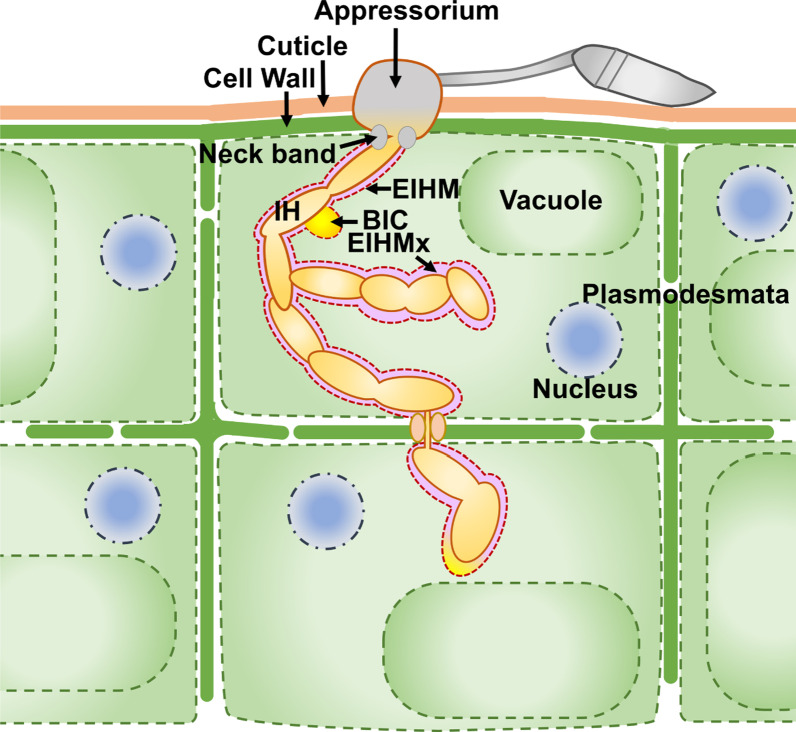
The infection strategy of *M. oryzae*. The spore of the fungi germinates and generates an appressorium. The appressorium penetrates the barriers of cuticle and cell wall, extending the invasive hyphae (IH), invaginating the plant plasma membrane. This plant plasma membrane covering the IH is known as the extrainvasive hyphal membrane (EIHM) and  the matrix between the plant and fungal plasma membranes forms the extrainvasive hyphal matrix (EIHMx). The first bulbous IH forms in the biotrophic invasion complex (BIC), which is the specialised region of EIHMx for fungal secretions. EIHMx forms the interface for interactions between the plant and fungi. The fungal IH continue to grow in the plant invading new cells and forming new BIC regions through plasmodesmata

The ascomycete fungus *M. oryzae,* causative agent of rice blast, is classified as one of the most devastating plant pathogens (Pennisi 2010; Dean et al. 2012). During the infection cycle, the fungal spore attaches to the leaf surface, germinates and the germ tube forms a specialised cell called appressorium, which develops the fungal hyphae and uses turgor pressure to insert the hyphae into the plant cell. The fungal hyphae invade plant tissues and cause necrotrophy, leading to disease lesions. *M. oryzae* gains access into the plant cytoplasm by inserting the invasive hyphae (IH) through the cell wall. The infection strategy of *M. oryzae* is outlined in Fig. 2.

The growing tips of primary IH and first bulbous IH retain the biotrophic invasion complex (BIC) which is the specialised region at primary IH for secretion of effectors (Khang et al. 2010; Yan and Talbot 2016). The BIC is a plant membrane-derived structure formed upon the invasion by fungus (Giraldo et al. 2013). Effector secretion at the BIC continues as the IH grow and branch in the plant cell. Through plasmodesmata the IH entering the neighbouring cell forms the BIC again. The BIC structure is a feature of successful infection and is not observed in resistant plants (Mosquera et al. 2009; Khang et al. 2010; Jones et al. 2016; Shipman et al. 2017).

Recently the fungal MAP Kinase, Pathogenicity MAP-Kinase 1 (PMK1) was shown to control the constriction of IH at the plasmodesmata to invade the neighbouring cells and regulate the expression of various effectors to suppress rice immune responses (Sakulkoo et al. 2018; Eseola et al. 2021). This secretion of a repertoire of effectors in a co-ordinated manner and with spatio-temporal dynamics plays a major role in *M. oryzae* infection.

### Apoplastic Effectors: ‘The Players at the Periphery’

**Fig. 3:**
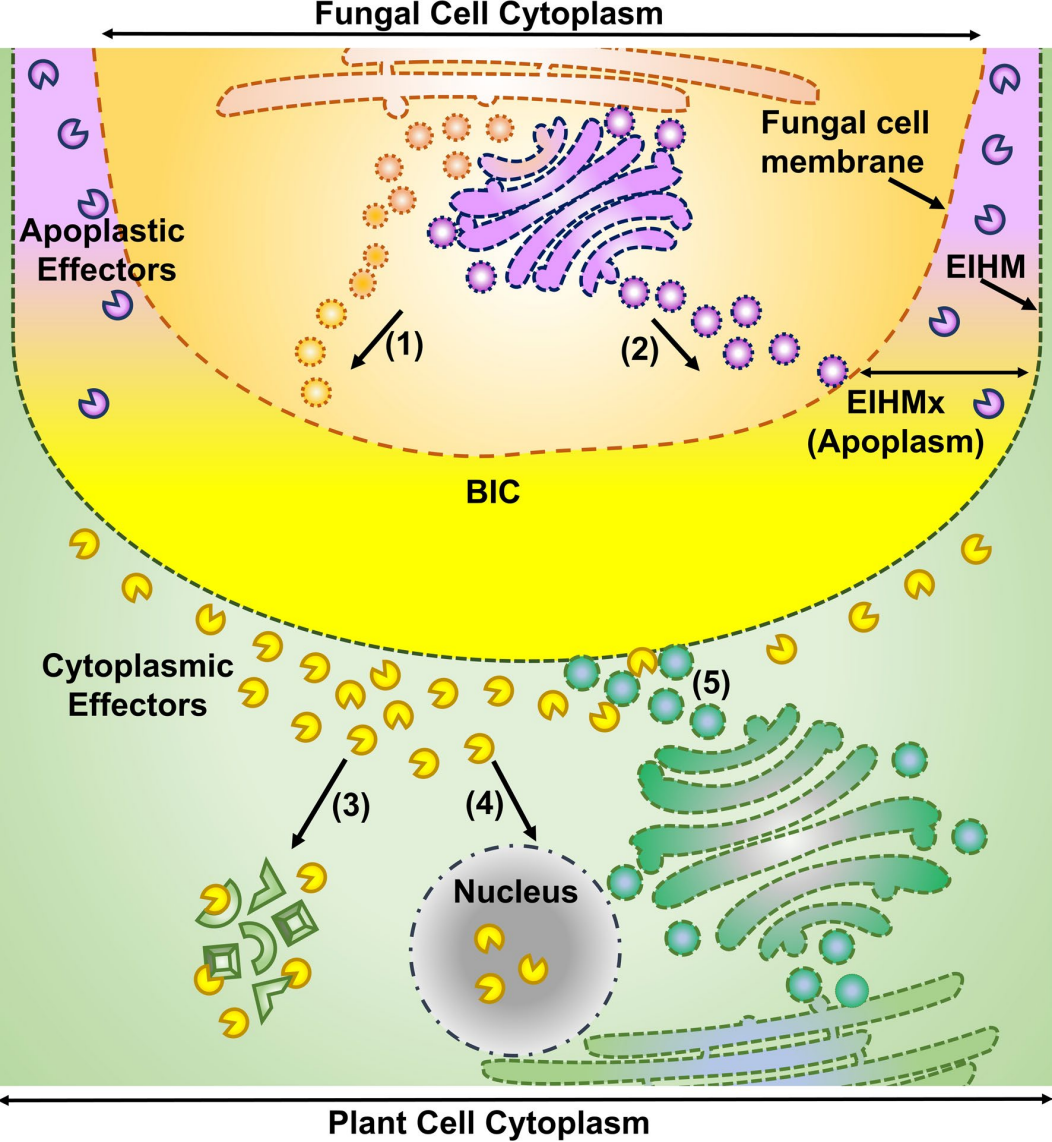
Effector secretory routes of *M. oryzae*. The fungal effector secretion takes place via vesicles and follows two routes. The BFA-insensitive vesicle secretion from endoplasmic reticulum (ER) which forms the BIC (1), which is destined for the plant cell cytoplasm, and the BFA-sensitive effector secretion, via Golgi apparatus, which is directed to the EIHMx (2). The effectors secreted to the EIHMx are the apoplastic effectors (violet pie shapes) and secretions from BIC are the cytoplasmic effectors (yellow pie shapes). The cytoplasmic effectors further localise to specific cell compartments such as the nucleus or plasmodesmata (3) or bind their target proteins (4), or have enzymatic activity, to compromise the plant cell and enable fungal growth. The plant cell cytoplasm is shown in green, bright yellow region represents BIC and BIC along with violet represents EIHMx or apoplasm

The effectors that remain in and are targeted to plant apoplast are known as apoplastic effectors. The apoplastic effectors follow a classical Golgi-dependant secretory pathway, which is sensitive to treatment by Brefeldin A (BFA). The apoplastic effectors are mostly localised in the extrainvasive hyphal matrix (EIHMx) lining the fungal cell (Giraldo et al. 2013) (Fig. 3). For example, among the apoplastic effectors, the biotrophy associated secreted (BAS) proteins have been described, which are a class of small cysteine-rich secreted proteins (Giraldo et al. 2013), some of which have been shown to localise in the EIHMx as well as at cell wall crossing points of IH (Mosquera et al. 2009; Khang et al. 2010).

Among the apoplastic effectors of *M. oryzae*, one functionally well-described effector is the **s**ecreted LysM (lysine motif) protein 1 (SLP1). Being an apoplastic effector, SLP1 accumulates in the EIHMx and competes with the rice pattern recognition receptor protein LysM protein, chitin elicitor binding protein (OsCEBiP), to bind chitin oligosaccharides and suppress chitin-induced immunity (Mentlak et al. 2012; Giraldo et al. 2013). Another apoplastic effector found to be competing with the OsCEBiP is MoAa91, a *M. oryzae* homolog of the auxiliary activity family 9 protein (Aa9) (Li et al. 2020).

### Cytoplasmic Effectors: ‘The Internal Intruders’

The cytoplasmic effectors have primarily been shown to be secreted and concentrated at the BIC and are eventually translocated to the plant cell cytoplasm. Some of the cytoplasmic effectors are also known to translocate into neighbouring cells via plasmodesmata (Khang et al. 2010). The cytoplasmic effectors follow a Golgi- independent secretory pathway involving the exocyst (Exo70 and Sec5) and t-SNARE (Sso1) complexes, via the endoplasmic reticulum. Hence, cytoplasmic effector secretion is BFA-insensitive (Giraldo et al. 2013) (Fig. 3).

Some examples include PWL2, Avr-Piz-t, and some BAS proteins (Mosquera et al. 2009; Khang et al. 2010). The cytoplasmic effector BAS107 has been shown to localise to the plant cell nucleus, suggesting a compartmental specialisation for the effectors. BAS1 and BAS2 were shown to preferentially localise to the BIC (Mosquera et al. 2009), and BAS107 and BAS1 translocated to uninvaded neighbouring plant cells via plasmodesmata (Khang et al. 2010). Among the small glycine-rich PWL (Pathogenicity toward Weeping Lovegrass) proteins, the cytoplasmic PWL1 and PWL2 have been shown to accumulate at the BIC, and PWL2 has been demonstrated to move from cell-to-cell via plasmodesmata (Sweigard et al. 1995; Kang et al. 1995; Khang et al. 2010).

One of the functionally well-characterised cytoplasmic effectors is Avr-Piz-t, which has been shown to interact with multiple proteins in the host (Li et al. 2009)*.* Avr-Piz-t has been shown to interact with a RING-domain E3 ubiquitin ligase, Avr-Piz-t Interacting Protein 6 (APIP6). The interaction leads to the ubiquitination of Avr-Piz-t and degradation of both Avr-Piz-t and APIP6, resulting in the suppression of PAMP-triggered immunity and increased susceptibility of rice (Park et al. 2012). The R gene, *Piz-t*, surprisingly does not have any direct interaction with the effector. Meanwhile, Piz-t is targeted for degradation by a second RING-domain E3 ubiquitin ligase, APIP10. APIP10 also interacts with Avr-Piz-t leading to its ubiquitination and degradation of both APIP10 and Avr-Piz-t. This degradation of APIP10 leads to stabilization of Piz-t and initiation of ETI (Park et al. 2016). The interaction of Avr-Piz-t with a bZIP transcription factor, APIP5, suppresses the function of APIP5 to promote effector-triggered necrotrophic cell death in rice (Wang et al. 2016a). A virulence target of Avr-Piz-t is the protein APIP12, a homologue of nucleoporin protein, Nup80, with which it interacts and reduces the basal resistance against *M. oryzae* (Tang et al. 2017). APIP4, a Bowman-Birk-type trypsin Inhibitor (BBI), interacts with Avr-Piz-t leading to a reduction in its trypsin inhibitor activity (Zhang et al. 2020). Avr-Piz-t also interacts with the Potassium (K^+^) channel protein OsAKT1, to suppress the rice innate immunity (Shi et al. 2018), and with the rice homologue of human small GTPase, OsRac1, to suppress the reactive oxygen species (ROS) production by the host (Bai et al. 2019).

Like Avr-Piz-t, another cytoplasmic effector Avr-Pii also interacts with more than one host protein and plays distinct roles in promoting pathogenesis. The interaction of rice exocyst complex protein OsExo70-F3 with Avr-Pii is necessary for immunity triggered by the cognate R protein, Pii (Fujisaki et al. 2015). Like other cytoplasmic effectors, Avr-Pii accumulates at the BIC and in rice cells, it interacts and inhibits the rice NADP-malic enzyme2 (Os-NADP-ME2). Inhibition of Os-NADP-ME2 reduces the NADPH levels, reducing the host ROS burst (Singh et al. 2016).

A family of structurally conserved fungal effectors has been described to share a conserved six β-sandwich structures with no significant sequence similarity. These effectors were named as MAX-effectors (*Magnaporthe* Avrs and ToxB-like), and include Avr-CO39, Avr-Pia, Avr-Piz-t, and ToxB (an effector of the wheat tan spot pathogen) (de Guillen et al. 2015). The Avr-CO39 effector has been shown to localise to the endoplasmic reticulum in the rice protoplast (Ribot et al. 2013), and is recognised by the NB-LRR protein pair, RGA4 & RGA5 (R-gene analog), which also recognize Avr-Pia. Both Avr-CO39 and AvrPia were shown to bind RGA5 (Cesari et al. 2013).

*M. oryzae* effectors have been found to affect multiple hormone signalling pathways as well. The *M. oryzae* hypothetical effector, MoHEG16 was shown to be necessary for the suppression of cell death caused by *M. oryzae* necrosis- and ethylene-inducing protein 1 (Nep1)-like proteins (MoNLPs) (Mogga et al. 2016). The interaction of *M. oryzae* cytoplasmic effector NIS1 with the rice receptor like kinase, OsBAK1, inhibits the kinase activity to suppress PTI (Irieda et al. 2019). IUG6 and IUG9 were identified as novel effectors, among other candidate genes in a new isolate of *M. oryzae*, showing a BIC localisation and suppression of salicylic acid and ethylene signalling (Dong et al. 2015).

Other than functional proteins, various metabolites or hormones have been shown to support the infection of *M. oryzae*. The enzyme, antibiotic biosynthesis monooxygenase (Abm), was shown to convert free jasmonic acid (JA) to Hydroxylated JA (12OH-JA), which helps the fungus to evade the rice immune responses. Abm localises to the fungal endoplasmic reticulum and BIC, indicating that Abm could be a secreted protein. Thus, both fungal derived enzymes and products of their activity together impart their action as effectors (Patkar et al. 2015). The avirulence conferring enzyme 1 (ACE1), an appressoria-localised effector protein, produces a secondary metabolite, which is the effector rather than the protein itself (Bohnert et al. 2004; Collemare et al. 2008). Like ACE1, the TAS1 enzyme has been shown to produce the well-characterised mycotoxin Tenuazonic acid (TeA) (Yun et al. 2015). Similarly, another enzyme, cytokinin synthesis 1, CSK1, was shown to be involved in active cytokinin production by *M. oryzae*. The cytokinin from fungus was shown to be involved in increasing metabolic availability, reducing defense responses and altering gene expression, thus suggesting that the cytokinin secreted by *M. oryzae* could be a classical effector (Chanclud et al. 2016). These studies implicate that the fungal secondary metabolites function as effectors and play key roles in disease progression. Table 3 summarises all the discussed *M. oryzae* effectors.

**Table 3:** Effectors of *M. oryzae*

Effector	Known function/related information	References
**Apoplastic effectors**		
SLP1	Competes with plant OsCEBiP to bind chitin oligosaccharides and helps the fungus suppress chitin-induced immunity in host; outlines IH, i.e. localised to EIHMx	Mentlak et al. (2012), Giraldo et al. (2013)
BAS3	focused point localisation in EIHMx & accumulates in the regions where IH cross at the cell wall to neighbouring cells	Mosquera et al. (2009)
BAS4	Outlines IH, i.e. localised to EIHMx	Mosquera et al. (2009)
BAS113	Outlines IH, i.e. localised to EIHMx	Giraldo et al. (2013)
MC69	Targeted gene disruption affects the pathogenicity of *M. oryzae*	Saitoh et al. (2012)
MSP1	Secreted into apoplasm; induces cell death & elicits immune responses	Wang et al. (2016c)
**Cytoplasmic effectors**		
PWL1	Accumulate at BIC, translocate to rice cytoplasm	Khang et al. (2010)
PWL2	Accumulate at BIC, translocate to rice cytoplasm, and move from cell to cell	Khang et al. (2010)
BAS1	Accumulate at BIC	Khang et al. (2010), Mosquera et al. (2009)
BAS2	Translocate to rice cytoplasm, and accumulate at cell wall crossing points	Mosquera et al. (2009)
BAS107	Accumulates at BIC, translocates and localises to rice cell nucleus, also moves from cell to cell	Giraldo et al. (2013)
Avr-Piz-t	Translocates to rice cells; interacts with Avr-Piz-t Interacting Protein 6 (APIP6, RING E3 ubiquitin ligase), APIP10 (RING E3 ubiquitin ligase), APIP5(bZIP transcription factor), APIP12 (homologue of nucleoporin protein, Nup80), OsAKT1 (Potassium (K^+^) channel protein) and OsRac1(homologue of human small GTPase) to suppress PTI	Park et al. (2012, 2016), Wang et al. (2016a), Tang et al. (2017), Shi et al. (2018), Bai et al. (2019)
Avr-Pii	Interact with OsExo70-F3 (exocyst complex protein) and Os-NADP-ME2 (NADP-malic enzyme2)	Fujisaki et al. (2015), Singh et al. (2016)
Avr-CO39	Translocates to rice cells; purified protein directly localises to protoplast without aid from fungal components, RAG5 interaction leads to recognition by RAG4/RAG5 R pair proteins	Ribot et al. (2013), Cesari et al. (2013)
Avr-Pia	RAG5 interaction leads to recognition by RAG4/RAG5 R pair proteins	Cesari et al. (2013)
MoHEG13	Suppresses the cell death caused by MoNLP proteins	Mogga et al. (2016)
MoHEG16	Necessary for successful virulence of *M. oryzae*	Mogga et al. (2016)
IUG6	BIC localisation and suppression of salicylic acid & ethylene signalling	Dong et al. (2015)
IUG9	BIC localisation and suppression of salicylic acid & ethylene signalling	Dong et al. (2015)
Avr-Pita	Predicted metalloprotease domain; binds to cognate R protein Pita directly; accumulates at BIC	Jia et al. (2000)
Avr-Pik/km/kp	The different alleles are pathogen race specific; have cognate functional R gene pair of NB-LRR with a set of *Pik* alleles in rice	Yoshida et al. (2009), Kanzaki et al. (2012)
Avr-Pi9	Localises to BIC and translocate to rice cells	Wu et al. (2015)
Avr-Pib		Zhang et al. (2015)
Avr-Pi54	Interacts directly with the R protein Pi54	Devanna et al. (2014)
Avr-Pi12		Li et al. (2018b)
**Secondary metabolites as effector**		
Hydroxylated Jasmonic acid (12OH-JA)	antibiotic biosynthesis monooxygenase (Abm) converts free jasmonic acid (JA) to Hydroxylated JA (12OH-JA)	Patkar et al. (2015)
Unknown secondary metabolite	Synthesis involves avirulence conferring enzyme 1, ACE1 an appressoria localised effector protein; the corresponding R gene is identified to be *Pi33*	Bohnert et al. (2004), Collemare et al. (2008)
Tenuazonic acid (TeA)	TAS1 is involved in the synthesis of TeA	Yun et al. (2015)
Cytokinin	Known protein involved is cytokinin synthesis 1, CSK1	Chanclud et al. (2016)

### Insect Pests

Rice is infested by a wide range of insect pests. The major insect pests include planthoppers, namely brown planthopper (BPH; *Nilaparvata lugens*), whitebacked planthopper (WBPH; *Sogatella furcifera*), smaller brown planthopper (SBPH; *Laodelphax striatellus*) and green rice leafhopper (GRH; *Nephotettix cincticeps*). Stemborers and Asian rice gall midge (*Orseolia oryzae*) are other major pests of rice (Bentur et al. 2016). Apart from damaging the crop by ingesting the phloem sap, many of these insects also transmit viruses that cause diseases in rice (Huang et al. 2019b).

**Fig. 4:**
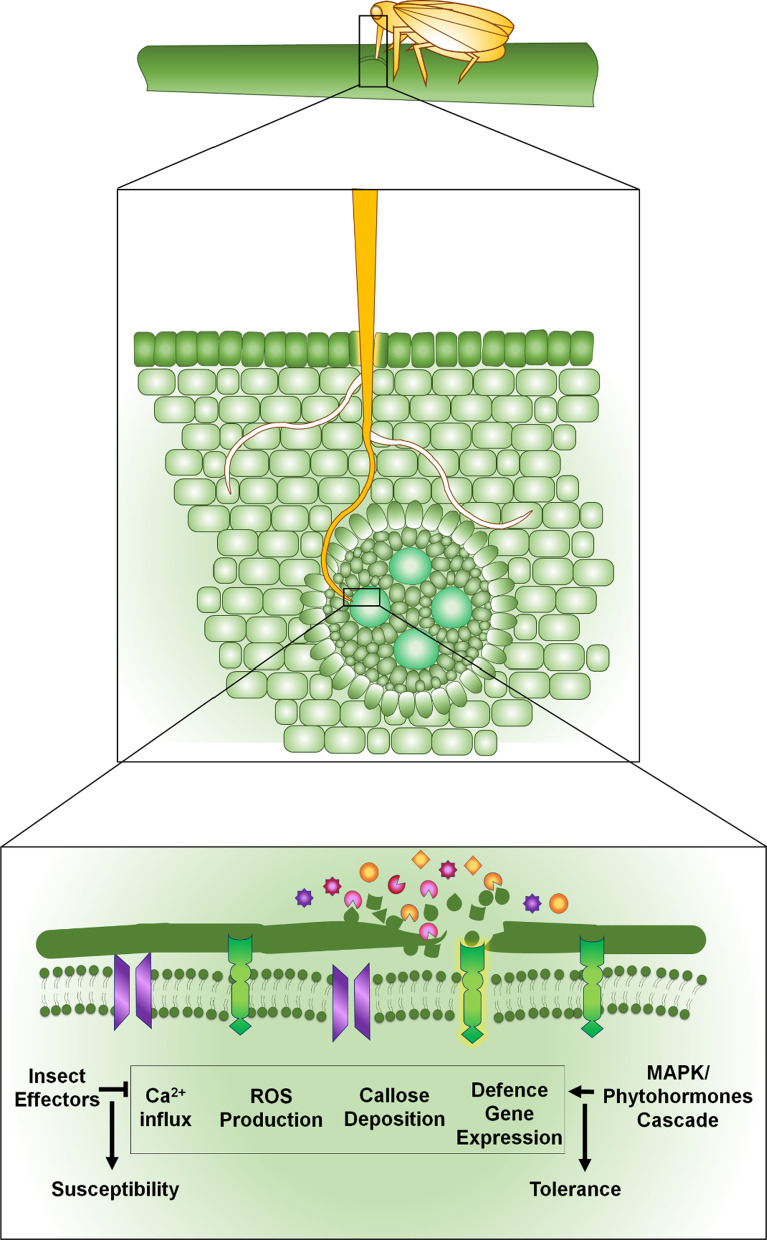
Schematic representation of BPH-Rice interaction. Using its stylet, the insect pierces the rice tissue and reaches the phloem to suck the sap. During this process, both gelling and watery saliva are secreted by the insect, which contain various molecules that elicit or act against plant defense. Proteins like catalase, endoglucanase, and Ca^2+^-binding proteins might be involved in suppressing the plant defense while proteins like Mucin-like proteins, Apolipophorins, and Protein disulfide isomerase elicit immune responses. Some of these proteins were found to induce callose deposition, cell death and SA or JA- associated defense gene expression

Different insects have different ways of obtaining their food. Among the piercing-sucking insects, BPH shows intracellular probing while GRH shows intercellular probing (Sōgawa 1982). The chewing insects access nutrients by causing mechanical damage to the host, whereas in piercing-sucking insects, the insect saliva forms the interface between the host and the insect. It has been shown that the insect saliva is composed of a diverse array of molecules. Majority of the studies on rice-insect interactions have been carried out with respect to the piercing-sucking insects like BPH and GRH. These insects probe the host tissue using their stylets in order to find a proper feeding site (Fig. 4). During this process, they secrete two types of saliva, the gelling saliva and the watery saliva. The gelling saliva is believed to aid in the production of a salivary sheath that might be helpful in providing mechanical strength to the stylet of the insect. Watery saliva is secreted into the plant tissue and might play a role in establishing proper conditions for accessing the nutrients, as has been established by various studies (Huang et al. 2019b). Hence, insect saliva is becoming an attractive area of study. Studies on plant- aphid interactions have demonstrated that the salivary components of the insect have the ability to alter the host physiology and also elicit the host response against the insect attack (Rodriguez and Bos 2013; Elzinga and Jander 2013).

Until now, many studies have demonstrated the global profile of the secretome of insects that attack rice, including BPH, WBPH, and GRH. Transcriptomics and proteomics approaches were used to profile the salivary gland transcriptome and the insect secretome, respectively. This section summarises these findings.

### Insect Saliva: A Repertoire of Diverse Set of Molecules

The role of insect saliva in the plant–insect interface has been known since the 1960s (Sogawa 1967). Studies on plant–insect interaction have revealed the ability of the insect saliva or oral secretions to induce and alter the host defense response (Rodriguez and Bos 2013; Acevedo et al. 2015). In rice, it was shown that the application of salivary gland extract of BPH causes rice transcriptional changes (Petrova and Smith 2015). Also, oral secretion of two chewing insects, viz., *Mythimna loreyi* and *Parnara guttata* were shown to elicit immune responses in rice (Shinya et al. 2016). These studies suggest that the salivary components of insects have the ability to alter the host physiology. Most of the studies in rice have been carried out with respect to the rice-planthopper interaction. Such studies have analysed the transcriptome or secretome of the insect salivary glands or saliva, respectively.

Transcriptomics studies of the salivary glands of BPH and GRH have established the global profile of the genes that are expressed in the insect salivary glands. Two different studies have identified 352 and 76 genes that encode secretory proteins in BPH and GRH, respectively (Ji et al. 2013; Matsumoto et al. 2014). Results from both these studies suggest that there are a large  number of proteins that are salivary gland-secreted, which function as enzymes. Among various predicted genes, those coding for serine protease, disulfide isomerase, lipase, and dehydrogenase were common with other piercing-sucking insects. In addition, the same study found that 45 out of the 68 salivary gland-specific transcripts code for unknown proteins (Matsumoto et al. 2014).

Other predicted genes encode for plant cell wall degrading enzymes, β-glucanases, β-glucosidases, glycosylases, trypsin-like proteins, lipases and α-amylase. Besides enzymes, genes encoding chemosensory proteins (CSPs) and odorant-binding proteins (OBPs) were also identified. It was previously shown in other systems that, CSPs and OBPs have host physiology altering ability (Ji et al. 2013). In addition, Ji et al. (2013) had identified a set of 67 salivary gland genes that are differentially expressed between two biotypes of BPH that differ in virulence. Another transcriptomics study identified 19 secretory proteins that might play a role in plant defense suppression and detoxification and digestion of the plant cell wall (Miao et al. 2018a).

Proteomics studies were crucial in establishing the truly secreted components of insect saliva. Approaches like 2D-PAGE and LC–MS/MS were used to study the insect saliva. All these studies used the saliva secretions of insects that were fed on artificial diet. In common, all the studies identified a wide variety of enzymes and proteins with  diverse functions to be present in the saliva of BPH, WBPH, and GRH (Konishi et al. 2009; Liu et al. 2016; Huang et al. 2016; Hattori et al. 2015; Miao et al. 2018b). Proteins involved in Ca^2+^-binding, ATP-binding, cytoskeletal, DNA- or RNA-binding, chromatin binding, transporters, apolipoproteins, ubiquitin, and heat shock proteins were also identified in the saliva (Liu et al. 2016). Details of the studies are shown in Table 4. These studies have established the components of insect saliva and laid the foundation for further studies on the molecular aspects of plant–insect interaction.

**Table 4:** Studies  that established the secretome of various rice pests

Insect	Source material	Approach	Number of proteins/genes identified	References
BPH	SG	2D-PAGE and Edman Degradation	52 proteins	Konishi et al. (2009)
BPH	Secreted Saliva	LC–MS/MS	202—Watery Saliva Proteins 18—Gelling Saliva Proteins	Huang et al. (2016)
BPH	Secreted Saliva	LC–MS/MS	107—Watery Saliva Proteins	Liu et al. (2016)
BPH	SG	Transcriptome	1140 genes coding secretory proteins	Rao et al. (2019)
BPH	SG	Transcriptome	352 genes coding secretory proteins	Ji et al. (2013)
BPH	SG	Transcriptome	19—SG Specific secreted protein encoding genes	Miao et al. (2018a)
GRH	Secreted Saliva	LC–MS/MS	71—Proteins	Hattori et al. (2015)
GRH	SG	Transcriptome	76 genes coding secretory proteins	Matsumoto et al. (2014)
WBPH	Secreted Saliva	LC–MS/MS	161—Watery saliva proteins	Miao et al. (2018b)

*BPH* Brown planthopper, *GRH* Green rice leafhopper, *WBPH* Whitebacked planthopper, *LC MS*/*MS Liquid Chromatography–Tandem Mass Spectrometry*, *SG* salivary gland, *2D-PAGE* two-dimensional polyacrylamide gel electrophoresis

### Insect Effectors: The Players at the Interface

Knockdown of insect genes using dsRNA has proved to be a valuable approach for functional characterisation of genes (Gu and Knipple 2013). Certain salivary proteins were found to be essential for the fitness of the insect. Knockdown of BPH *NlMul* (*Nilaparvata lugens Mucin-like protein*) has resulted in short and single-branched stylets. *NlMul* gene codes for a mucin-like protein that is present in abundance in the insect saliva. Mucins are highly glycosylated proteins and are important for the cell-environment communication (Huang et al. 2017). Knockdown of another salivary protein-encoding gene in BPH, *NlShp* (*Nl Sheath protein*) was shown to inhibit the formation of salivary flanges and salivary sheath. NlShp was annotated as an unknown protein (Huang et al. 2015). Another study revealed that three protein coding genes, including an annexin-like protein (*ANX-like 5*), a salivary sheath protein (*salivap-3*) and a carbonic anhydrase (*CA*), are essential for the survival of BPH in rice. Further *ANX-like 5* and *salivap-3* were shown to be indispensable for the feeding behaviour of BPH wherein the knockdown of these genes showed negative effects on phloem sap-feeding time and honeydew excretion by BPH (Huang et al. 2016). These studies support the notion that proper salivary sheath formation is essential for BPH virulence in rice. In GRH, knockdown of *NcSP75,* (*Nephotettix cincticeps* *Salivary Protein 75kDa*)encoding a salivary protein of unknown function, was shown to cause poor performance of the insect on rice, while no such effects were seen in insects raised on artificial diet (Matsumoto and Hattori 2018). As the knockdown of these genes led to lesser virulence of the insect, it is possible that these gene products aid in the interaction between the host and the pest.

In order to reach the vascular bundle, the insect uses its stylet to probe and pierce through the plant tissue. During this process, the insect has to break the cell wall components of the plant cell. The expression of genes coding for plant cell wall degrading proteins in the BPH salivary gland was described previously (Ji et al. 2013). *NlEG1*, a predicted endo-β-1,4-glucanase, was shown to have in-vitro endoglucanase activity and the knockdown of *NlEG1* reduced the insect’s ability to reach the phloem and also had negative impacts on food intake, mass, survival, and fecundity of the insect on rice plants. Additionally, only a small effect on survival was seen in the insects that were raised on artificial diet. Hence, it is speculated that NlEG1 might act as an effector which alters the host structures and enables the stylet to reach the phloem (Ji et al. 2017).

Further, catalase gene named *Kat-1* was shown to be secreted into the rice tissue and possess catalase activity (Petrova and Smith 2014). It is speculated that Kat-1 might be helpful in scavenging the hydrogen peroxide (H_2_O_2_) molecules released by the plant post insect attack. A mucin-like protein, NlMLP was also shown to be important for insect performance and salivary sheath formation. In addition, NlMLP was found to induce cell death when transiently expressed in either rice protoplasts or *Nicotiana benthamiana* leaves. The induction of cell death was found to be calcium-dependent and acting through the MEK2-dependent MAPK pathway. Also, NlMLP was shown to induce callose deposition and trigger jasmonic acid-related defense gene expression in *N. benthamiana* (Shangguan et al. 2018).

Calcium signalling is known to be an immediate response by plants after insect attack. It results in the occlusion of sieve elements, thereby preventing the insects from ingesting the sap (Rodriguez and Bos 2013). But the insects are mostly successful in overcoming this block. Studies had shown that insect saliva possesses Ca^2+^-binding proteins. NlSEF1 is an EF-hand Ca^2+^-binding protein present in the saliva of BPH and is secreted into the rice tissue. Also, it was shown that NlSEF1 reduces the cytosolic Ca^2+^ levels in rice and suppresses wound-induced H_2_O_2_ production. Moreover, the knockdown of *NlSEF1* decreased the survival and feeding of the insects (Ye et al. 2017). A similar protein was also characterised in GRH. NcSP84 was found to be a salivary protein that exhibits in-vitro Ca^2+^-binding activity and is secreted into the rice tissue (Hattori et al. 2012). The putative effectors thus far identified have been tabulated in Table 5. These studies support the possible role of insect-associated molecules in suppressing the plant defense response.

**Table 5:** Insect associated molecules that are characterised

Insect	Protein	Description	Activity and localisation in rice	References
BPH	NlMLP	Mucin-like protein	Cell death and callose deposition Cytoplasm	Shangguan et al. (2018)
BPH	NlSEF1	EF-hand Ca^2+^-binding protein	Suppression of wound-induced H_2_O_2_ and reduction in cytosolic Ca^2+^ level	Ye et al. (2017)
BPH	NlEG1	endo-β-1,4-glucanase	Possesses in vitro endoglucanase activity	Ji et al. (2017)
BPH	NlMul	Mucin-like protein	–	Huang et al. (2017)
BPH	Kat-1	Catalase	In vitro catalase activity	Petrova and Smith, (2014)
BPH	salivap-3	Salivary Protein	–	Huang et al. (2016)
BPH	CA	Carbonic Anhydrase	–	Huang et al. (2016)
BPH	ANX-like 5	Annexin-like protein 5	–	Huang et al. (2016)
BPH	N112	Protein disulfide isomerase	Cell death/Nucleo-cytoplasmic	Rao et al. (2019)
BPH	N116	Apolipophorin-III	Cell death/Nucleo-cytoplasmic	Rao et al. (2019)
BPH	N128	Small secreted cysteine-rich protein	Cell death/Nucleo-cytoplasmic	Rao et al. (2019)
BPH	N132	Chemosensory protein	Nucleo-cytoplasmic	Rao et al. (2019)
BPH	N140	Unknown Protein	–	Rao et al. (2019)
BPH	N143	Unknown protein	Cell death/Nucleus	Rao et al. (2019)
GRH	NcSP75	Unknown Protein	–	Matsumoto and Hattori, (2018)
GRH	NcSP84	EF-hand Ca^2+^-binding protein	In vitro Ca^2+^ binding activity	Hattori et al. (2012)
GRH	β-glucosidase	β-glucosidase	In vitro hydrolysis of p-nitrophenyl-b-d-glucopyranoside	Nakamura and Hattori, (2013)
GRH	NcLac1S	Laccase	In vitro laccase activity	Hattori et al. (2010)
SBPH	DNaseII	Deoxyribonuclease II	In vitro DNAse activity; suppression of insect-induced callose and H_2_O_2_ accumulation	Huang et al. (2019a)
RGM	OoNDPK	Nucleoside diphosphate kinase	Secreted into the host cells; causes elongation of rice coleoptile cells	Sinha et al. (2012)

*BPH* Brown Planthopper, *GRH* Green Rice Leafhopper, *SBPH* Small Brown Planthopper, *RGM* Rice Gall Midge

In order to identify the effector properties of a candidate protein, transient transformation of *N. benthamiana* followed by cell death assays is widely used. Using such a strategy, six putative effectors were identified in BPH after screening 64 candidates. The six putative effectors include protein disulfide isomerase (PDI; N112), apolipophorin (N116), small secreted cysteine-rice protein (SSCP; N128), chemosensory protein (CSP; N132), and two proteins with no predicted functions (N140 and N143). These proteins were found to induce cell death, chlorosis, or dwarf phenotype in *N. benthamiana*. In addition, the proteins also induced defense responses including callose deposition and defense gene expression (Rao et al. 2019). Cellular damage could occur during the penetration of insect stylet which in turn would result in the release of cellular components. The presence of deoxyribonucleic acid (DNA) in the extracellular region is also known to trigger plant defense responses by acting as a damage-associated molecular pattern (DAMP) (Quintana-Rodriguez et al. 2018). Previous studies have reported the presence of deoxyribonuclease II (DNase II) in the saliva of planthoppers (Liu et al. 2016; Miao et al. 2018b). The DNase II in small brown planthopper saliva suppresses induction of plant defense responses including H_2_O_2_ accumulation and callose deposition. In addition, it was shown that the exogenous application of DNase II slightly reduced those responses (Huang et al. 2019a).

Another pest, the rice gall midge (RGM), causes gall formation in susceptible rice varieties and is a major threat for crop production. RGM induces gall formation in rice apical meristem by altering the rice metabolic pathways in order to facilitate its own survival (Sinha et al. 2011). Although a serious pest of rice, the studies on the pest effectors that are involved in establishing gall formation are somewhat limited. In one study, gene expression analysis of RGM maggots identified a nucleotide diphosphate kinase (*NDPK*), that is highly expressed in compatible interaction than in incompatible interaction (Sinha et al. 2012). NDPK was identified to be secreted into rice during RGM feeding and the application of recombinant NDPK resulted in the elongation of rice coleoptile cells. This study suggested a possible role of NDPK in facilitating the alteration of host machinery to establish gall formation by RGM.

Many rice resistance genes have been identified against the major rice pests including the planthoppers and the gall midge. Like many disease resistance genes, the insect resistance genes also encode Nucleotide binding-site-Leucine rich repeat-containing proteins (NBS-LRRs), among others (Bentur et al. 2016; Fujita et al. 2013). This suggests that a direct recognition of the pest-associated molecules may be occuring in the cytoplasm of plant cells.

## Conclusions: Diverse Attackers—Common Pathways

The pathogens and pests discussed in this review represent a diverse group of organisms but with rice as a common host. The first striking difference between the different pathogens and pests is the mode of effector secretion. In bacteria, dedicated type II and type III secretion systems are involved in the secretion of effector proteins, whereas fungal pathogens employ the BFA- sensitive or BFA- insensitive vesicular pathways for effector secretion (Jha et al. 2007; Giraldo et al. 2013). Insects, on the other hand, secrete saliva, which contains the complete repertoire of effectors (Shangguan et al. 2018). Pathogenesis begins by the invasion of the plant cell. This is accomplished by the apoplastic effectors, which breach the plant cell wall and facilitate entry into the host cellular system. Most of these apoplastic effectors have defined enzymatic activity, the majority of which are directed towards disruption of cell wall barriers leading to nutrient availability (Ji et al. 2013, 2017; Jha et al. 2005; Tayi et al. 2016b; Rajeshwari et al. 2005; Aparna et al. 2009; Zou et al. 2012).

However, for suppression of plant immune responses, these different pathogens and pests seem to target common nodes in plant defense. Some of the common pathways targeted by the pathogens and pests include well-characterised immune response components, such as the MAPK pathway, ubiquitination pathway, calcium signalling, and hormone signalling. For example, both bacterial peptidoglycan and fungal chitin are recognised by the OsCERK1 receptor complex, which further phosphorylates the cytoplasmic receptor kinase OsRLCK185 and activates MAPK cascades (Akamatsu et al. 2013; Wang et al. 2017; Yamaguchi et al. 2013b; Ao et al. 2014). This seems to be a critical step in the induction of defense responses against multiple pathogens, and hence is also a target for suppression of immune responses by the pathogens, eg., by XopY_Xoo_, XopP_Xoo_, MoSLP1, and Avr-Piz-t (Yamaguchi et al. 2013b; Mentlak et al. 2012; Giraldo et al. 2013; Bai et al. 2019; Ishikawa et al. 2014). The MAPK signalling pathway amplifies the plant immune responses, thus making it another nodal point for suppression. Numerous effector proteins target the MAPK signalling events, hence modulating the plant immune responses (Long et al. 2018; Mentlak et al. 2012; Giraldo et al. 2013). In parallel, some of the pathways which are activated early on during pathogen infection and pest infestation include calcium signalling and the oxidative burst (Akamatsu et al. 2013), which pathogens and pests have evolved to suppress in order to cause infection (Giraldo et al. 2013; Mentlak et al. 2012; Bai et al. 2019; Singh et al. 2016). For instance, an effector from BPH, NlSEF1, suppresses cytosolic Ca^2+^ levels and wound- induced H_2_O_2_ (Ye et al. 2017), whereas the *Magnaporthe* effectors Avr-Piz-t and Avr-Pii were shown to suppress ROS levels (Bai et al. 2019; Singh et al. 2016). Another important molecular cascade that is targeted by pathogens to evade immune activation is regulation via ubiquitination, specifically, by targeting the E3 Ubiquitin ligases, which regulate the final step of ubiquitin conjugation (Ishikawa et al. 2014; Park et al. 2016, 2012).

The hormone signalling pathways are important targets for host defense manipulation. Effectors from pathogens and pests modulate components of hormone pathways to suppress the plant defenses. Effectors from *Xoo* have been shown to suppress immune responses by targeting brassinosteroid signalling (Yamaguchi et al. 2013a; Wang et al. 2008), whereas *Magnaporthe* effectors modulate cytokinin and active jasmonic acid levels *in planta* (Chanclud et al. 2016; Patkar et al. 2015).

Yet another common feature among effector proteins is functional compartmentalisation. Some of the bacterial non-TAL effectors as well as the TAL effectors are known to translocate to the nucleus in plant cells. Although the precise functions have not been elucidated for fungal and pest effectors, nuclear localisation is observed, suggesting that nuclear localisation is possibly important for the function of several effectors (Gurlebeck et al. 2006; Mosquera et al. 2009; Giraldo et al. 2013; Deb et al. 2019, 2020; Rao et al. 2019). In bacterial pathogens, some TAL effectors specifically target and regulate gene expression of susceptibility factors like *SWEET* genes (Yang et al. 2006; Antony et al. 2010; Yuan et al. 2009). This seems to be crucial in *Xoo* since geographically distant strains of *Xoo* were shown to upregulate the same or different *SWEET* genes through different TAL effectors (Streubel et al. 2013).

The primary requirement of the pathogen and pest is immune evasion in order to establish itself in the host. For this, they target multiple pathways in rice. The convergence of effector functions could be attributed in part to the common host pathways which are involved in defense against multiple pathogens and pests. The characterisation of such key components of the plant immune system would lead to a more comprehensive understanding of plant resistance responses to pathogens and pests.

## Future Perspectives

Although numerous studies have been focused on understanding the mechanism of host- pathogen/pest interactions in rice, there is a lot that remains to be explored. Gaps in our knowledge exist regarding the molecular mechanisms of effector action in rice. It is established that different pathotypes of a pathovar or biotypes of an insect possess an effectome repertoire specific for causing disease in a plant genotype in a particular geographical location. How this diversity in effectome and the crosstalk between the effectors helps in disease development remains to be determined. Studies on hub proteins in immune signalling pathways are also crucial to understand immune response functioning. Ultimately, this knowledge should be leveraged to develop crop varieties that are resistant to multiple pathogens and pests, thus helping to meet the increasing demand of global rice production.

## Data Availability

Data sharing is not applicable to this article as no datasets were generated during this study.

## Abbreviations

12OH-JA: Hydroxylated JA
2D-PAGE: Two-dimensional polyacrylamide gel electrophoresis
BAS: Biotrophy associated secreted
BB: Bacterial Blight
BBI: Bowman-Birk-type trypsin Inhibitor
BFA: Brefeldin A
BIC: Biotrophic invasion complex
BLS: Bacterial Leaf Streak
BPH: Brown planthopper
CWDE: Cell Wall Degrading Enzymes
DAMP: Damage-Associated Molecular Pattern
DNA: Deoxyribonucleic acid
EIHM: Extrainvasive hyphal membrane
EIHMx: Extrainvasive hyphal matrix
ETI: Effector triggered immunity
GRH: Green rice leafhopper
IH: Invasive hyphae
JA: Jasmonic acid
LC MS/MS: Liquid Chromatography–Tandem Mass Spectrometry
*M. oryzae*: *Magnaporthe oryzae*
NADPH: Nicotinamide adenine dinucleotide phosphate
NBS-LRR: Nucleotide-Binding Site-Leucine Rich Repeats
PAMP: Pathogen-Associated Molecular Pattern
PGN: Peptidoglycan
pv.: Pathovar
PWL: Pathogenicity toward Weeping Lovegrass
RGA: R-gene analog
R-gene: Resistance gene
RGM: Rice Gall Midge
RNA: Ribonucleic acid
ROS: Reactive oxygen species
SBPH: Small Brown planthopper
SG: Salivary Gland
T2SS: Type 2 Secretion System
T3SS: Type 3 Secretion System
TALE: Transcription-activator like effector
TeA`: Tenuazonic acid
WBPH: White-backed planthopper
*Xoc*: *Xanthomonas oryzae* pv. *oryzicola*
*Xoo*: *Xanthomonas oryzae* pv. *oryzae*
Xop: Xanthomonas outer protein
